# Implementing a nationwide criteria-based emergency medical dispatch system: A register-based follow-up study

**DOI:** 10.1186/1757-7241-21-53

**Published:** 2013-07-09

**Authors:** Mikkel S Andersen, Søren Paaske Johnsen, Jan Nørtved Sørensen, Søren Bruun Jepsen, Jesper Bjerring Hansen, Erika Frischknecht Christensen

**Affiliations:** 1 Research Department, Prehospital Emergency Medical Services, Aarhus, Central Denmark Region, Olof Palmes Allé 34, Aarhus N 8200, Denmark; 2 Department of Clinical Epidemiology, Aarhus University Hospital, Aarhus, Denmark; 3 Emergency Medical Communication Center, Copenhagen, Capital region of Denmark; 4 Emergency Medical Communication Center, Odense University hospital, Odense, Region of Southern Denmark

**Keywords:** Emergency medical dispatch, Criteria-based dispatch, Emergency medical services, Case fatality risk, Implementation

## Abstract

**Background:**

A criteria-based nationwide Emergency Medical Dispatch (EMD) system was recently implemented in Denmark. We described the system and studied its ability to triage patients according to the severity of their condition by analysing hospital admission and case-fatality risks.

**Methods:**

This was a register-based follow-up study of all 1-1-2 calls in a 6-month period that were triaged according to the Danish Index – the new criteria-based dispatch protocol. Danish Index data were linked with hospital and vital status data from national registries. Confidence intervals (95%) for proportions with binomial data were computed using exact methods. To test for trend the Wald test was used.

**Results:**

Information on level of emergency according to the Danish Index rating was available for 67,135 patients who received ambulance service. Emergency level A (urgent cases) accounted for 51.4% (n = 34,489) of patients, emergency level B for 46.3% (n = 31,116), emergency level C for 2.1% (n = 1,391) and emergency level D for 0.2% (n = 139). For emergency level A, the median time from call receipt to ambulance dispatch was 2 min 1 s, and the median time to arrival was 6 min 11 s. Data concerning admission and case fatality was available for 55,270 patients. The hospital admission risk for emergency level A patients was 64.4% (95% CI = 63.8-64.9). There was a significant trend (p < 0.001) towards lower admission risks for patients with lower levels of emergency. The case fatality risk for emergency level A patients on the same day as the 1-1-2 call was 4.4% (95% CI = 4.1-4.6). The relative case-fatality risk among emergency level A patients compared to emergency level B–D patients was 14.3 (95% CI: 11.5-18.0).

**Conclusion:**

The majority of patients were assessed as Danish Index emergency level A or B. Case fatality and hospital admission risks were substantially higher for emergency level A patients than for emergency level B–D patients. Thus, the newly implemented Danish criteria-based dispatch system seems to triage patients with high risk of admission and death to the highest level of emergency. Further studies are needed to determine the degree of over- and undertriage and prognostic factors.

## Background

Emergency medical dispatch (EMD) systems aim to match response resources with patient needs. However, the organization of EMD systems varies substantially across healthcare systems, and there is no consensus regarding the optimal organization [1]. Emergency medical service calls are typically handled by an emergency medical communication centre (EMCC), which assesses the urgency of the call in order to determine the priority level of the response. Depending on country or area, the EMCC can be staffed by lay-persons that have received some training or by firemen, paramedics, nurses and doctors. EMD is usually carried out in accordance with a predefined framework of instructions, with Criteria-Based Dispatch (CBD) and Medical Priority Dispatch System® (MPDS) being the most widespread.

In Denmark, the handling of all out-of-hospital medical emergencies has recently been reorganized. This was done by a nationwide introduction of EMCCs and the implementation of a criteria-based dispatch protocol termed the Danish Index for Emergency Care (Danish Index). Danish EMCCs are staffed by nurses, paramedics and doctors who assess and prioritize 1-1-2 calls. These tasks were previously performed mainly by the police. All Danish residents have free access to health care, including emergency medical services (EMS) and hospitals as a tax-financed service.

We aimed to describe the new Danish emergency medical dispatch system. Accordingly, this paper reports the first data on the distribution of the levels of emergency of 1-1-2 calls and the corresponding prehospital time intervals. We also aimed to investigate the EMD systems ability to triage patients according to severity, by using admission risk and case fatality risk as proxies for severity of patient condition.

## Methods

### Setting

In Denmark, the 1-1-2 emergency number is used for all emergencies, including those that require police-, fire- and health-related responses. All 1-1-2 calls are answered by the police or fire brigade. In mid-2011, five regional EMCCs were introduced in Denmark to provide EMD service to the entire country. The assessment and prioritization of citizens with medical problems who called the 1-1-2 number was done previously by the police (or, in part of the capital, by the fire brigade). After determining the caller’s location, the 1-1-2 operator now transfer all health-related calls to the appropriate EMCC where the calls are assessed. The EMCC staff determines the level of emergency and decides on a response using the Danish Index, a criteria-based dispatch protocol for assessing the calls, making decisions about the emergency level and determining the appropriate responses [2,3]. The Danish Index has 37 main symptom groups that are each subdivided into five levels of emergency; each level of emergency contains a number of more specific symptoms. The five levels of emergency are as follows: A: life-threatening or potentially life-threatening condition, immediate response required; B: urgent, but not life-threatening condition; C: non-urgent condition that needs an ambulance; D: non-urgent supine patient transport; and E: other service or advice/instruction including taxi transportation (no ambulances are dispatched for emergency level E calls). The Danish Index also suggests supplementary questions to ask the caller and advice for lay bystanders and for health care professionals. 1-1-2 calls that are answered by an EMCC are assigned a Danish Index criteria code that corresponds to the level of emergency, main symptom and specific subgroup symptom.

### Population and study design

We conducted a register-based follow-up study of all patients that contacted an EMCC through the 1-1-2 number. Data were collected during the last 6 months of 2011 from three of the five regional EMCCs in Denmark. The combined population of the three regions (the Capital Region of Denmark, the Central Region of Denmark and the Region of Southern Denmark) is 4,165,361 inhabitants, representing approximately 75% of the total Danish population [4]. The Capital Region consists of mainly urban areas with a population density of 665 inhabitants/km^2^. The Central and Southern Regions of Denmark include both urban and rural areas and have lower population densities of 99 and 96 inhabitants/km^2^, respectively [4].

### Data sources and variables

The EMCC dispatch software was used to identify all 1-1-2-related assignments. The study variables extracted from the dispatch software for each assignment included the patient’s civil registration number, the Danish Index code and prehospital time intervals. The prehospital time intervals obtainable from the EMCC software included the EMD response interval and the EMS response interval as defined in Utstein style by Castren et al. [5]. The EMD response interval is the time from registration of a call by the EMCC software until activation of the first responding ambulance. The EMS response interval is the time from activation of the first ambulance until its arrival on scene.

In order to retrieve additional follow-up data we utilized the fact that each Danish citizen is assigned a unique 10-digit civil registration number. This number is used in all Danish registries and enables unambiguous linkage among these registries [6]. If a patient had no civil registration number registered in the EMCC software we were unable to retrieve register-based follow-up data. For this study we used two national registries, the Danish Civil Registration System (CRS) and the Danish National Registry of Patients (NRP). The NRP was established in 1977 and has records of all Danish hospital visits and admissions. The registry includes information on numerous variables, including civil registration number, dates of hospital admission and discharge and discharge diagnoses classified according to the Danish version of the WHO’s International Classification of Diseases, 10^th^ edition (ICD-10). The NRP has tracked 99.4% of all discharges from Danish acute care non-psychiatric hospitals since 1977 and all hospital outpatient and emergency department visits since 1995 [7]. For this study, the hospital admission date and the discharge date were retrieved from the NRP.

The Danish Civil Registration System was established in 1968 and registers all persons living in Denmark [6]. For this study we retrieved data on gender, date of birth and changes in vital status (dead or alive) from the CRS. The vital status data was used to calculate case fatality risk.

### Statistics

The outcomes included the Danish Index level of emergency, the main index group, the EMD and EMS response intervals, admission to hospital and death (within 24 h, 48 h and 30 days after the 1-1-2 call). Proportions were reported with 95% confidence intervals (95% CI) computed as CIs for proportions with binomial data using exact methods. Rates per 1,000 were assumed to follow a Poisson distribution and 95% CIs were computed according to that. Relative risk (RR) estimates were calculated as risk ratios comparing emergency level A patients with combined emergency level B through D and RR estimates are presented with the 95% CI. Time intervals were reported as medians with interquartile range (IQR). We used the Wald test to test for trends. All analyses were performed using STATA statistical software, version 12.

### Ethics

The study was approved by the Danish Data Protection Agency. According to Danish law, permission from the Ethics Committee or from individual patients is not required for register-based studies.

## Results

**Figure 1:**
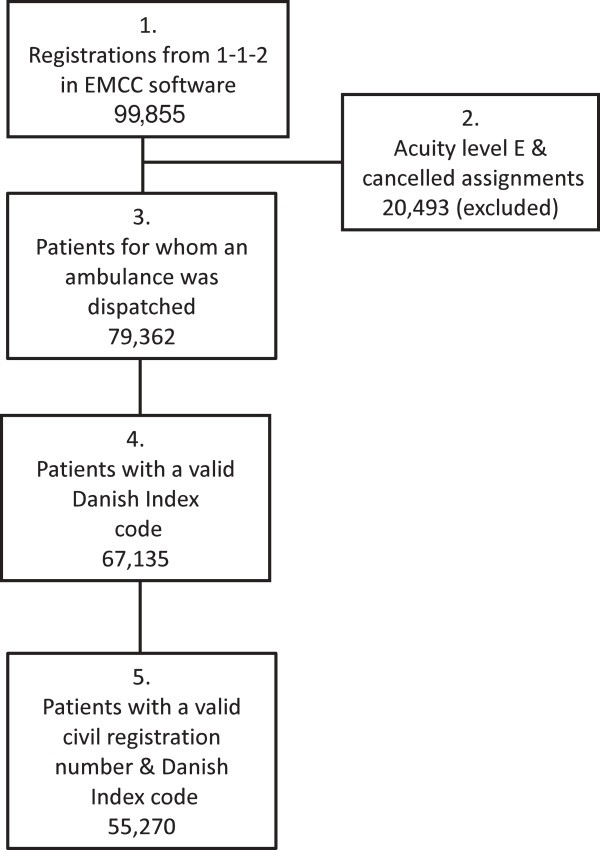
**Included patients and patients lost to follow-up due to incomplete registration.** Box **1**: Total number of 1-1-2 registrations in the study period. Box **2**: Excluded patients. Emergency level E and cancelled assignments. Box **3**: All patients that received an ambulance via a 1-1-2 call. Box **4**: Patients, that received an ambulance via a 1-1-2 call, with a valid Danish Index code registered. Box **5**. Patients that received an ambulance via a 1-1-2 call, with a valid Danish Index code *and* a valid civil registration number registered (making complete follow-up possible).

In the six-month study period from July 2011 to December 2011, a total of 99,855 1-1-2-related registrations were identified in the three included EMCCs. Of these, 20,493 did not lead to dispatch of an ambulance, mainly because they were assessed as emergency level E or were cancelled for other reasons (e.g. multiple calls regarding the same incident). A total of 79,362 ambulances were dispatched i.e. one ambulance per patient. A valid Danish Index code was registered for 67,135 of these, a valid Danish Index code *and* a valid civil registration number was available for 55,270 (70%) of the patients receiving an ambulance (Figure 1). The mean patient age was 54.9 years, and 47.7% were female and 52.3% male. Of the patients receiving an ambulance in the study period, 81.0% appeared in the dataset only once.

Out of the total 67,135 patients with a valid Danish Index code registered, emergency level A accounted for 51.4% (n = 34,489) , emergency level B patients for 46.3% (n = 31,116), emergency level C patients for 2.1% (n = 1,391) and emergency level D patients for 0.2% (n = 139). That corresponds to a total rate of 32.2 ambulance turnouts per 1,000 inhabitants per year in the three regions. The capital region had 32.9 turnouts per 1.000 inhabitants, the southern region 36.4 and the central region had 27.3. The rates of turnouts per 1.000 inhabitants are shown on a regional level in Table 1. The five most frequently used Danish Index main symptom groups were: 1) unclear problem; 2) chest pain, heart disease; 3) minor wound, fracture or injury; 4) accident (not traffic-related); and 5) difficulty in breathing. The distribution of the main symptom groups according to level of emergency is shown in Table 2.

**Table 1:** Number of patients receiving an ambulance per 1,000 inhabitants per year in the three included regions

	**All N = 67,135**	**Capital (n = 28,030)**	**Central (n = 17,233)**	**Southern (21,872)**
	**(95% CI)**	**(95% CI)**	**(95% CI)**	**(95% CI)**
A	16.6 (16.38-16,74)	13.1 (12,86-13,34)	16,7 (16,38-17,02)	21.3 (20,94-21,68)
B	14.9 (14,78-15,1)	19.4 (19,06-19,66)	10,4 (10,12-10,62)	13.5 (13,18-13,78)
C	0.7 (0,64-0,7)	0.5 (0,40-0,5)	0.2 (0,16-0,24)	1.5 (1,38-1,58)
D	0.07 (0,06-0,08)	0.02 (0.2-0,04)	0.04(0,04-0,06)	0.2 (0,12-0,2)
All	32.2 (32.0-32,48)	32.9 (32,54-33,32)	27.3(26,9-27,72)	36.4 (35,94-36,92)

The study contains data from a 6 months period, the estimates in this table has been extrapolated to one year.

**Table 2:** Level of emergency and main symptom groups

**Main Index Group**	**All (%)**	**A (%)**	**B (%)**	**C (%)**	**D (%)**
Unclear problem	11,534 (17.1)	3,909 (11.3)	7,396 (23.8)	214 (15.4)	15 (10.8)
Chest pain, heart disease	8,737 (13.0)	7,661 (22.2)	1,018 (3.3)	56 (4,0)	2 (1.5)
Minor wound, fracture, injury	7,373 (11.0)	423 (1.2)	6,494 (20.9)	384 (27.6)	72 (51.8)
Accident (not traffic related)	6, 490 (9.6)	2,116 (6.1)	4,141 (13.3)	210 (15.1)	23 (16.6)
Difficulty in breathing	4,945 (7.3)	3,341 (9.7)	1,433 (4.6)	170 (12.2)	1 (0.7)
Impaired consciousness, paralysis	4,464 (6.6)	4,051 (11.8)	377 (1,2)	35 (2.5)	1 (0.7)
Poisoning, medications, alcohol, drugs	3,962 (5.9)	1,204 (3.5)	2,704 (8.7)	53 (3.8)	1 (0.7)
Seizure	3,420 (5.1)	1,794 (5.2)	1,626 (5,2)	.	.
Traffic accident	3,145 (4.6)	2,373 (6.9)	762 (2,5)	.	10 (7.2)
Stomach or back pain	2,950 (4.4)	659 (1.9)	2,175 (7.0)	115 (8.3)	1 (0.7)
Unconscious adult	2,342 (3.4)	2,339 (6.8)	3 (0.0)	.	.
Bleeding–non traumatic	1,227 (1.8)	689 (2.0)	494 (1.6)	44 (3.2)	.
Diabetes	1,149 (1.7)	594 (1.7)	533 (1.7)	21 (1.5)	1 (0.7)
Psychiatry, suicide	1,017 (1.5)	539 (1.6)	476 (1.5)	2 (0.1)	.
Allergic reaction	758 (1.1)	582 (1.7)	176 (0.6)	.	.
Violence, abuse	522 (0.8)	216 (0.6)	304 (1.0)	1 (0.1)	1 (0,7)
Sick child	476 (0.7)	391 (1.1)	84 (0.3)	1 (0.1)	.
Gynaecology, pregnancy	435 (0.7)	259 (0.8)	142 (0.5)	34 (2.4)	.
Headache	414 (0.6)	384 (1.1)	9 (0.0)	21 (1.5)	.
Ear, nose, throat	278 (0.4)	66 (0.2)	202 (0.7)	10 (0.7)	.
Urinary system	273 (0.4)	12 (0.0)	255 (0.8)	6 (0.4)	.
Fire or electricity injury	248 (0.4)	144 (0.4)	103 (0.3)	1 (0.1)	.
Fever	182 (0.3)	127 (0.4)	55 (0.2)	.	.
Foreign body in airway	145 (0.2)	131 (0.4)	14 (0.0)	.	.
Childbirth	120 (0.2)	96 (0.3)	13 (0.0)	.	11 (7.9)
Possible death or Sudden Infant Death	93 (0.2)	76 (0.2)	16 (0.0)	1 (0.1)	.
Eye	83 (0.2)	37 (0.1)	41 (0.1)	5 (0.4)	.
Unconscious child	82 (0.1)	82 (0.2)	.	.	.
Animal and insect bites	74 (0.1)	57 (0.2)	13 (0.0)	4 (0.3)	.
Hypo- and hyperthermia	64 (0.1)	42 (0.1)	22 (0.1)	.	.
Chemicals and gases	54 (0.1)	32 (0.1)	20 (0.1)	2 (0.1)	.
Drowning	34 (0.1)	30(0.1)	4 (0.0)	.	.
Poisoning in children	27 (0.0)	17 (0.1)	10 (0.0)	.	.
Skin and rash	10 (0.0)	8 (0.0)	1 (0.0)	1 (0.1)	.
Diving accident	5 (0.0)	5 (0.0)	.	.	.
Large scale accident	3 (0.0)	3 (0.0)	.	.	.
**All**	**67,135 (100)**	**34,489 (100)**	**31,116 (100)**	**1,391 (100)**	**139 (100)**

All patients with a valid Danish Index code (67.135) distributed according to main symptom group and level of emergency, as defined by The Danish Index for Emergency Care.

The overall median EMD response interval was 2 min 34 s. The median EMD response interval for the most urgent emergencies within all main symptom groups was 1 min 46 s. For the most severe main symptom category (A.01.01), “unconscious, not breathing normally”, a group containing the majority of suspected cardiac arrests, the median EMD response interval was 1 min 34 s (mean, 2 min 5 s). The median EMS response interval for emergency level A patients was 6 min 11 s, and 75% of all emergency level A turnouts arrived on scene within 9 min 17 sec. Table 3 shows the EMD and EMS response intervals according to emergency level.

**Table 3:** Emergency medical dispatch and emergency medical services response intervals in minutes and seconds

**Emergency**	**No.**	**EMD, median (IQR)**	**EMS, median (IQR)**
**Level**			
A	34,489	02:01 (1:28,2:47)	6:11 (4:18,9:17)
B	31,116	03:27 (2:20,5:38)	10:00 (6:50,14:24)
C	1,391	04:51 (3:00,10:41)	11:14 (07:44,17:27
D	139	6:46 (3:37,19:00)	13:00 (8:33,21:07)
All	67,135	02:34 (1:45,4:01)	7:53 (5.09,11:59)

Interquartile range (IQR).

EMD response interval: Time spend from reception of 1-1-2 call at the EMCC, until activation of the ambulance. EMS response interval: Time spend from ambulance activation until arrival on scene. Patients with a valid Danish Index code (67,135) are included in the table.

Follow-up data on admission to hospital and vital status (dead or alive) were available for the 55.270 patients with both a Danish Index code *and* a civil registration number registered. The admission risks among emergency level A and D patients were 64.4% (95% CI = 63.8-64.9) and 31.2% (95% CI = 22.7-40.8), respectively. The relative risk (RR) of admission among emergency level A patients compared to emergency level B, C and D patients combined was 1.25 (95% CI = 1.23-1.27). Admission risk data is shown in Table 4. Patients not admitted to the hospital as inpatients received either sufficient treatment on-scene (by EMS staff or prehospital physician), or received treatment for minor injuries in the Emergency Department and then sent home. Among emergency level A patients with complete follow-up data available, the case fatality risk on the same date as the 1-1-2 call was 4.4% (95% CI = 4.13-4.60), and the risk increased to 8.6% (95% CI = 8.28-8.94) after 30 days. Among emergency level B patients, the case fatality risk on the same date as the 1-1-2 call was 0.3% (95% CI = 0.23-0.37), and the risk increased to 3.3% (95% CI = 3.09-3.55) after 30 days. Emergency level A patients had a relative risk of dying of 14.3 (95% CI: 11.5-18.0) the same day as the 1-1-2 call compared to levels B through D combined. The case fatality risks and RR of death are shown in Table 5.

**Table 4:** Admission to hospital risk for patients in the indicated Danish Index emergency level groups

**Emergency level**	**No.**	**Admitted to**	**Admission risk,**
		**hospital**	**% (95% CI)**
A	28,630	18,440	64.4 (63.8-65.0)
B	25,419	13,190	51.9 (51.3-52.5)
C	1,112	475	42.7 (39.8-45.7)
D	109	34	31.2 (22.7-40.8)
**All**	**55,270**	**32,139**	58.1 (57.7-58.6)*

*Test for trend, p < 0.001.

All patients with Danish Index code and civil registration number (55.270) registered (a prerequisite for follow-up data from national registries). Patients hospitalized for one day or more are regarded as admitted.

**Table 5:** Case fatality risk for patients in the indicated Danish Index emergency level groups

**No.**	**0–24 h (95% CI)**	**0–48 h (95% CI)**	**30-day (95% CI)**
A 28,630	4.4 (4.13-4.60)	5.1 (4,87-5,39)	8.6 (8.28-8.94)
B 25,419	0.3(0.23-0.37)	0.6 (0.47-0.66)	3.3 (3.09-3.55)
C 1,112	0.4 (0.15-1.05)	0.5 (0.20-1.17)	3.3 (2.35-4.56)
D 109	0 (0–3.32)*	0 (0–3.32)*	0.9 (0.02-5.0)
RR A vs. B-D	14.3 (11.5-17.98)	9.2 (7.80-10.92)	2.6 (2.42-2.81)
**All 55,270**	**2.4 (2.28-2.54)**	**2.9 (2.78-3.07)**	**6.1 (5.85-6.25)**

*One-sided, 97.5% confidence interval.

Case fatality risk for patients in the indicated Danish Index emergency level groups and the relative risk (RR) of dying for group A patients compared to group B, C and D patients combined. Analysis based on 55.270 patients with Danish Index code *and* civil registration number registered (a prerequisite for follow-up data from national registries).

## Discussion

This study showed that the majority of 1-1-2 callers in contact with the Danish EMCCs were assessed as being Danish Index emergency level A or B. The symptoms reported most frequently by callers were unclear problem, chest pain, minor wounds and injuries, accidents and difficulties in breathing. Both the EMD and EMS response intervals were shortest for emergency level A patients. Admission and case-fatality risks were substantially higher for emergency level A patients than for emergency level B–D patients.

The new EMD system enables linkage between dispatch data and patient outcome data. The previous system, which was staffed by police, did not register each patient’s civil registration number; therefore there are no comparable Danish data at the individual level before the introduction of EMCCs. Comparisons with results reported by other EMD systems are warranted, but hampered by several factors. First, EMD organization differs considerably in different countries. Secondly, uniform guidelines for EMD reporting have only been available for a few years [5,8].

Kuisma et al. reported on an EMD system in the Helsinki area of Finland [9]. In a four emergency levels system, the distribution of calls according to emergency was: A, 5.7%; B, 27.0%; C, 47.4%, and D, 19.9%. These numbers are quite different from ours; however, the differences can probably be explained by the fact that not only assignments originating from 1-1-2 calls, but also other ambulance requests were included in the Finnish study.

Norway has an EMD system and a prehospital organization that is very similar to the recently implemented Danish system. In a study by Zakariassen et al. of the Norwegian Index, which served as the basis of the Danish Index, they found a rate of emergency level A turnouts in Norway of 25 per 1,000 inhabitants per year. [10] That is a higher rate than the 16.6 in our study. A part of the difference can be explained by missing data in our study. Based on our data the rates per 1,000 inhabitants underestimates by approximately 18%. Some of the regional differences in rates per 1.000 inhabitants observed in our study can be explained by differences in demographics between the regions. Zakariassen et al. also reported a patient distribution in the main symptom groups similar to our findings. Specifically, for emergency level A, the five most frequent main symptoms were chest pain (22%), patient transport (ordered by hospitals and general practitioners) (18%), unclear problem (14%), accidents and traffic accidents (12%) and unconscious adult or child (8%). The corresponding emergency level A data in our study (Table 2) were chest pain (22.2%), accidents and traffic accidents (13%), unclear problem (11.3%) and unconscious adult or child (7.0%). Patient transports ordered by hospitals and general practitioners were not a part of our study since they are not handled by the Danish 1-1-2 system. The Danish Index is a new feature of a very young organization, which may explain the high proportion of patients with unclear problems in our study. When a serious condition is suspected, health care personnel probably tend to rapidly deploy the desired response team rather than spending time determining the relevant main symptom group. However, the similar finding of a high proportion of unclear problems in Norway, where the criteria-based EMD organization is well established, identifies a possible inadequacy in the Danish and Norwegian Indexes. In a study in the US, Sporer et al. found breathing problems reported in 12.2% of all calls, chest pain in 6.0%, unclear problems in 1.1%, seizures in 3.4%, falls in 8.7% and fainting/unconsciousness in 8.7% [11]. Sporer et al. reported on an MPD system that uses slightly different main symptom groups; nevertheless, the small proportion of unclear problems and the high proportion of breathing problems stands out compared to our results. Other studies of MPD systems have typically reported unclear problems in 5–8% of patients [12,13] The fixed algorithm structure of the MPDS may explain some of the difference in the proportion of breathing problems. In Denmark all citizens have 24 hr access to a general practioner, which may also explain some of the differences.

The median EMD response interval for potential cardiac arrests (unconscious, not breathing normally) was 1 min 34 s (mean, 2 min 5 s) in our study. For 373 known out-of-hospital ventricular fibrillation cases in Finland, Kuisma et al. found an EMD response interval of 77.1 ± 44 s [14]. In an EMD system resembling the Danish CBD system, Berdowski et al. examined the handling of out-of-hospital cardiac arrests in the Amsterdam area [15]. They found a mean EMD response interval for suspected cardiac arrests of 1.88 minutes (1 min 53 s), a result similar to our findings. These results raise the question of whether this amounts to a fast or a slow processing of calls concerning potentially serious emergencies. A recent *Circulation* editorial stated that high performance Medical Priority Dispatch Systems typically have vehicles rolling ≤ 30 seconds from call receipt [16]. Compared with our > 90 seconds, this seems very fast. Since EMD systems aim to balance response resources with patient needs, it is worth considering whether a short EMD response interval in itself is an indicator of high quality in dispatching. Except for cardiac arrest where a quick dispatch is of major importance, the time spent clarifying the situation may help uphold high quality dispatching. Data regarding the time interval from a 1-1-2 call is received by the police and until it is passed on to the EMCC was not available for this study.

The EMS response interval, which is often described as the ambulance response time, is a topic that receives much attention from researchers, health care professionals, administrators, politicians and the general public. Many EMS systems have a target response time of less than 8 minutes for acute response. There is robust evidence for an association between short EMS response interval and increased survival only for cardiac arrest patients [17]. In a study of North American trauma patients with field-based physiological abnormalities, Newgard et al. found no association between the response time (or other prehospital time interval) and mortality. For 3,656 ambulance dispatches they reported an impressive median EMS response interval of 4.28 min with an IQR of 3.0–6.3 min [18]. In a study in the US, Pons et al. reported a median EMS response interval of 5.8 min (IQR 4.3–7.7 min) [19]. Many North American studies are conducted in areas that include very large cities. The three regions included in our study contained a mixture of urban and rural areas with different locally-determined target values for EMS response intervals. The target values concerning acuity level A turnouts was a median of 8 min in one region, a mean of 10 min in another and 75% below 10 min in the third region. All regional target values were met during the study period.

The hospital admission risk was highest among emergency level A patients and correlated directly with emergency level. Specifically, we found a clear trend of lower admission risk for lower levels of emergency (Table 4). If we consider admission risk to be a proxy for the severity of the patient’s condition, this trend indicates that the new Danish EMD system triages severely ill patients appropriately. A similar trend was found in a Canadian study by Blanchard et al. in which 7,603 of 23,442 (32.4%) of lower emergency level patients were admitted as inpatients and 3,141 of 7,943 (39.5%) of higher emergency level patients were admitted as inpatients [20]. In our study, a similar pattern was observed regarding case fatality risk, which was much higher among patients assessed as emergency level A compared with patients assessed as having a lower emergency level (Table 5). In a Finish study, Kuisma et al. observed a similar trend in the prehospital case fatality risk, which was of 5.2% among emergency level A patients and 1.1% for level B, 0.1% for level C and 0.03% for level D patients [9].

We had no data on the physiological status of the patients at the time of ambulance arrival on scene or upon arrival at the hospital. Precise estimates of over- and undertriage in terms of sensitivity, specificity and predictive values were therefore not possible to make. However our results do allow considerations about triage precision. The results regarding admission to hospital suggests a degree of overtriage among emergency level A patients of about 35%, since their condition could be treated on scene or in the emergency department. On the other hand, a part of the 35% non-admitted emergency level A patients, may have been in severe distress, but treated sufficiently on scene or in the emergency room. A part of the admitted patients in the lower emergency level groups may represent undertriage, especially the emergency level B and C patients dying on the same day as the 1-1-2 call may represent undertriage. But the case fatality risks in these groups are quite small, indicating that undertriage is not extensive. In all systems some degree of mistriage is unavoidable. When looking at e.g. trauma patients, the American College of Surgeons states that 5–10% undertriage is probably inevitable and overtriage of 30–50% is common in trauma-triage systems [21]. Some quantity of overtriage is definitely needed to avoid oversights of severe conditions.

The strengths of our study include the population-based design and its representation of 75% of the Danish population. Other strengths include the large study volume, which allowed statistically precise estimates and the ability to follow-up patients to determine hospital admission and case-fatality risks. One limitation is that a part of the patients had missing data due to incomplete registration of either the Danish Index code, civil regis-tration number or both. The entry of index codes and civil registration numbers into the EMCC software is based mainly on manual typing by the EMCC staff. This is a large part of the explanation for the missing data. There are also situations where patients are unable to inform their civil registration number, or the caller is a third party with no knowledge about patient identity. Other reasons for missing civil registration numbers are foreign patients, patients unwilling to inform identity and oversights by EMCC or EMS staff. When looking at the rate of missing data in smaller clusters (e.g. comparing the three EMCCs, comparing shorter time periods) we found no indications of selection bias. The missing registration of about 15% of all Danish Index codes makes our results regarding rates of turnouts per 1,000 inhabitants underestimates of the true values.

## Conclusions

Using case fatality and hospital admission risks as indicators of case severity, the new Danish criteria-based dispatch system seems to triage patients with high risk of admission and death to the highest level of emergency. Further studies are needed to determine the degree of over- and undertriage and studies of the Danish Index as a predictor of death or severe illness and injury are warranted.

## Abbreviations

CBD: Criteria-based dispatch; CI: Confidence interval; CRS: Civil registration system; EMCC: Emergency Medical Communication Centre; EMD: Emergency medical dispatch; EMS: Emergency medical services; IQR: Interquartile range; MPDS: Medical priority dispatch system; NRP: National registry of patients; RR: Relative risk.

## Competing interests

The authors declared that they have no competing interests.

## Authors’ contributions

MSA, SPJ and EFC have designed the study. MSA, JNS, SBJ, JBH have made substantial contributions to the acquisition of data. All authors have contributed substantially in the analysis and interpretation of data, drafting the manuscript and have given final approval of this version.
